# Low serum magnesium levels are associated with increased risk of fractures: a long-term prospective cohort study

**DOI:** 10.1007/s10654-017-0242-2

**Published:** 2017-04-12

**Authors:** Setor Kwadzo Kunutsor, Michael Richard Whitehouse, Ashley William Blom, Jari Antero Laukkanen

**Affiliations:** 1 Musculoskeletal Research Unit, School of Clinical Sciences, University of Bristol, Learning and Research Building (Level 1), Southmead Hospital, Southmead Road, Bristol, BS10 5NB UK; 2 Institute of Public Health and Clinical Nutrition, University of Eastern Finland, Kuopio, Finland; 3 Internal Medicine, Central Finland Central Hospital, Jyväskylä, Finland

**Keywords:** Magnesium, Cohort study, Fracture, Risk factor

## Abstract

Magnesium, which is an essential trace element that plays a key role in several cellular processes, is a major component of bone; however, its relationship with risk of major bone fractures is uncertain. We aimed to investigate the association of baseline serum magnesium concentrations with risk of incident fractures. We analyzed data on 2245 men aged 42–61 years in the Kuopio Ischemic Heart Disease prospective cohort study, with the assessment of serum magnesium measurements and dietary intakes made at baseline. Hazard ratios [95% confidence intervals (CI)] for incident total (femoral, humeral, and forearm) and femoral fractures were assessed. During a median follow-up of 25.6 years, 123 total fractures were recorded. Serum magnesium was non-linearly associated with risk of total fractures. In age-adjusted Cox regression analysis, the hazard ratio (HR) (95% CIs) for total fractures in a comparison of the bottom quartile versus top quartile of magnesium concentrations was 2.10 (1.30–3.41), which persisted on adjustment for several established risk factors 1.99 (1.23–3.24). The association remained consistent on further adjustment for renal function, socioeconomic status, total energy intake, and several trace elements 1.80 (1.10–2.94). The corresponding adjusted HRs for femoral fractures were 2.56 (1.38–4.76), 2.43 (1.30–4.53) and 2.13 (1.13–3.99) respectively. There was no evidence of an association of dietary magnesium intake with risk of any fractures. In middle-aged Caucasian men, low serum magnesium is strongly and independently associated with an increased risk of fractures. Further research is needed to assess the potential relevance of serum magnesium in the prevention of fractures.

**Electronic supplementary material:**

The online version of this article (doi:10.1007/s10654-017-0242-2) contains supplementary material, which is available to authorized users.

## Introduction

Magnesium is an essential trace element that plays a key role in several cellular processes including nucleic acid synthesis, enzymatic reactions, and cell replication. It is also an important component of bone, with 67 percent of total body magnesium known to be found in the bone tissue [1]. A number of factors are known to play an important role in bone health and these include ageing, heritability, sex, physical activity, hormonal factors and nutrition [2].

Investigations of the effect of nutrition on bone health has mostly focused on specific dietary factors such as calcium and vitamin D. There have been suggestions that magnesium might also be linked with bone heath. In both human and animal experimental models, magnesium deficiency has been shown to be associated with decreased osteoclastic and osteoblastic activity, osteopenia and skeletal fragility [3, 4].

Although it appears that magnesium may have a beneficial effect on bone health, its relationship with fractures is uncertain. It has been postulated that magnesium may be involved in the development of fractures via (1) modulation of osteoclastic and osteoblastic activity; [5] (2) its effects on bone density and fragility; [3] and (3) alteration in levels of other micronutrients such as calcium and vitamin D [1, 5], which are needed for bone health. A number of epidemiological as well as clinical studies have reported dietary magnesium intake and magnesium supplementation to be linked to greater bone mineral density [6–9], suggesting that magnesium may be associated with risk of fractures. In an analysis of the Norwegian Epidemiologic Osteoporosis Studies (NOREPOS) hip fracture database, Dahl et al. [10] demonstrated a protective association between magnesium in drinking water and risk of hip fractures. However, a recent meta-analysis of 12 studies, has demonstrated that high intakes of magnesium were not associated with risk of hip and total fractures [11]. Given the inconsistent evidence on the association between dietary magnesium and fracture risk and the dependence of serum magnesium concentrations on dietary magnesium sources as well as water [12, 13], it would be clinically useful to ascertain if serum magnesium concentrations are linked to fracture risk. Data however on the association between serum magnesium status and risk of major fractures is sparse. In a longitudinal analysis of the European Prospective Investigation into Cancer and Nutrition (EPIC)–Norfolk cohort, Hayhoe et al. demonstrated statistically significant trends between serum magnesium concentrations and risk of fractures; however, the associations were attenuated to null after adjustment for confounding factors [14]. Fractures (particularly osteoporotic fractures) are one of the leading worldwide causes of disability and morbidity, especially among the aging population, and increase the burden on health systems [15]. The prevention of fractures is therefore of public health importance and the evaluation of putative risk factors such as serum magnesium, which may have predictive or causal relevance to the risk of fractures and which could help tailor preventive and therapeutic interventions is a priority. In this context, our first objective was to assess the shape, nature, and magnitude of the prospective association between serum magnesium with risk of incident total and femoral fractures. The second objective was to assess the consistency of the association in important clinical subgroups using a population-based cohort of 2245 relatively healthy men from eastern Finland. In subsidiary analysis, we assessed the association of dietary magnesium intake with the risk of fractures in the same set of participants.

## Methods

This report was conducted according to STROBE (STrengthening the Reporting of OBservational studies in Epidemiology) guidelines for reporting observational studies in epidemiology (online Appendix 1) [16].

### Study population

The Kuopio Ischaemic Heart Disease (KIHD) population-based prospective cohort analyzed in this study was set up primarily to investigate risk factors for cardiovascular disease and other additional health outcomes in eastern Finland [17]. This cohort has been described in detail previously; [17–19] in brief, the KIHD cohort consisted of a representative sample of 3433 randomly selected men aged 42–61 years living in the city of Kuo-pio and its neighbouring rural communities who participated in the baseline study conducted between March 1984 and December 1989. Of the 3433 men, 3235 were found to be eligible; and of this number, 2682 (78%) volunteered to participate, 186 did not respond to the invitation, and 367 declined to give informed consent. The data set analyzed in the present study includes 2245 men with baseline data on serum magnesium, relevant covariates, and fracture outcomes. The ethical committee of the University of Eastern Finland approved all procedures involving human subjects and each study participant provided written informed consent according to the Declaration of Helsinki.

### Ascertainment of incident fractures

All incident fractures, representing all femoral, humeral, and forearm fracture cases that occurred from enrollment to 2014 were included. The primary outcome measures included incident total and femoral fractures. No losses to follow-up were recorded. In the KIHD study, participants are under annual continuous surveillance for the development of new outcome events, including fractures [20]. Fracture incidence data were collected from the National Hospital Discharge Register data by computer linkage and a comprehensive review of hospital records, discharge diagnoses, and inpatient physician claims. The events were coded according to the International Classification of Diseases Tenth Revision diagnostic codes for fractures by site.

### Measurement of risk factors

Methods for collection of blood specimens and the measurement of lipids, biochemical analytes, and all trace elements have been previously described in detail [21]. Briefly, besides fasting overnight before blood collection, participants were told to abstain from drinking alcohol for at least 3 days and from smoking for at least 12 h before assessment. Serum samples collected were stored frozen at −80 °C for 0.2–2.5 years. Serum magnesium was measured using atomic absorption spectrometry (Perkin Elmer Zeeman 5000, Perkin Elmer, Norwalk, CT, USA) which involved the use of acetylene-air (1:4) flame technique. Serum magnesium was diluted in a ratio of 1:50 with distilled water. The wavelength was 185.2 nm for magnesium. The between-run Coefficient of Variation for the method was 2.4% (37 assays) [22]. The measurement of pH-corrected serum ionized calcium concentrations was made using ion selective electrodes (Microlyte 6, Kone, Finland; CV 1.6%). Measurement of serum zinc concentrations were made using the PerkinElmer 306 atomic absorption spectrophotometer (Norwalk, Connecticut, USA). Participants completed self-administered health and lifestyle questionnaire for the assessment of age, smoking, alcohol consumption, socio-economic status (SES), baseline diseases, and medical history [21]. Energy expenditure of physical activity was assessed using the validated KIHD 12-month leisure-time physical activity questionnaire [23, 24]. Dietary magnesium, energy, and other nutrient intakes were assessed quantitatively at the time of blood sampling by recording food intake over 4 days using a validated questionnaire [25]. During dietary assessment, common sets of household measures were used to help the participants assess food portion sizes. Instructions were given and completed food records were checked by an experienced nutritionist. The average of the 4 days dietary intake was calculated. Food intakes were converted into nutrient intakes using the Nutrica software (version 2.5; National Public Health Institute, Turku, Finland), which uses mainly Finnish values of nutrient composition of foods and takes into account loss of vitamins during food preparation. The Nutrica software contains a large database comprising 1300 food items and dishes and the nutrient composition of foods, including dietary magnesium. The Nutrica software was developed at the Research Center of the Social Insurance Institution of Finland.

### Statistical analysis

Baseline characteristics were presented as means (SD) or median (interquartile range) for continuous variables and percentages for categorical variables. Cross-sectional correlations of serum magnesium levels with various risk markers were determined by calculating partial correlation coefficients adjusted for age. Cox proportional hazard regression models were used to conduct time-to-event analyses. Schoenfeld residuals were used to confirm the proportional hazards assumptions [26]. To characterize the shape of the association between serum magnesium and risk of total fractures, hazard ratios (HRs) were calculated within quartiles of serum magnesium concentrations and plotted against the mean serum magnesium concentrations within each quartile. Floating variances were used to calculate 95% confidence intervals for the log hazard ratio in each group, including the reference group, to allow for comparisons across the groups irrespective of the arbitrarily chosen reference category (top quartile) [27]. As the association showed a non-linear shape, HRs were calculated by quartiles defined according to the baseline distribution of serum magnesium concentrations. Hazard ratios were progressively adjusted for (1) age; (2) plus body mass index (BMI), height, systolic blood pressure (SBP), smoking status, history of diabetes mellitus, and physical activity; and (3) plus estimated glomerular filtration rate (eGFR), as calculated using the Chronic Kidney Disease Epidemiology Collaboration formula [28], SES, energy intake, serum zinc, serum potassium, and serum ionized calcium. These confounders were selected based on their previously established role as risk factors for fractures, evidence from previous research, or their potential as confounders based on known associations with fracture outcomes and observed associations with serum magnesium using the available data [29]. Collinearity diagnostics using the variance inflation factor [30] showed no evidence of collinearity between BMI and height. We evaluated effect modification by pre-specified clinically relevant characteristics using interaction tests. To avoid potential bias due to participants at high risk of fractures at baseline, we carried out sensitivity analyses that excluded the first 5 years of follow-up. All statistical analyses were conducted using Stata version 14 (Stata Corp, College Station, TX, USA).

## Results

### Baseline characteristics and correlates of serum magnesium levels

Table 1 summarizes characteristics of the study population (n = 2245). The mean (SD) serum magnesium levels and dietary magnesium intake at baseline were 1.98 (0.15) mg/dl and 417.2 (69.3) mg/day respectively. Based on the suggested normal reference range of 1.8–2.3 mg/dl for serum magnesium [31], 136 (6.1%) men had hypomagnesemia (<1.8 mg/dl) and only 22 (1%) men had excess serum magnesium levels (>2.3 mg/dl) in the study population. The mean (SD) age and BMI of study participants were 53 [5] years and 26.9 (3.6) kg/m^2^ respectively. Serum magnesium levels were weakly and inversely correlated with alcohol consumption, SES, fasting glucose, and renal function. Weak positive correlations were observed for age, total cholesterol, creatinine, zinc, and serum ionized calcium. No significant correlation was observed with dietary magnesium intake. Online Appendix 2 shows baseline characteristics of study participants by quartiles of serum magnesium. Except for a few characteristics, there were generally no significant differences in baseline characteristics across quartiles of serum magnesium.

**Table 1:** Baseline participant characteristics and correlates of serum magnesium

	Overall (N = 2245) Mean (SD), median (IQR), or n (%)	Partial correlation, r (95% CIs)^†^
Magnesium (mg/dl)	1.98 (0.15)	–
*Questionnaire/prevalent conditions*		
Age at survey (years)	53.1 (5.0)	0.05 (0.01–0.09)*
Alcohol consumption (g/week)	75.9 (137.6)	−0.08 (−0.13 to −0.04)***
Total energy intake (kJ/day)	9855 (2609)	−0.03 (−0.07 to 0.01)
Socioeconomic status	8.51 (4.24)	−0.06 (−0.10 to −0.02)*
Dietary magnesium intake (mg/day)	417.2 (69.3)	−0.03 (−0.07 to 0.01)
History of diabetes		
No	2156 (96.0)	–
Yes	89 (4.0)	–
Smoking status		
Other	1529 (68.1)	–
Current	716 (31.9)	–
History of hypertension		
No	1564 (69.7)	–
Yes	681 (30.3)	–
*Physical measurements*		
BMI (kg/m^2^)	26.9 (3.6)	−0.02 (−0.06 to 0.02)
Height (cm)	172.8 (6.2)	−0.00 (−0.04, 0.04)
SBP (mmHg)	134 (17)	−0.03 (−0.07 to 0.01)
DBP (mmHg)	89 (10)	−0.01 (−0.05 to 0.03)
Physical activity (kj/day)	1546 (1489)	0.00 (−0.04 to 0.04)
*Lipid markers*		
Total cholesterol (mmol/l)	5.90 (1.08)	0.07 (0.03–0.11)***
HDL-C (mmol/l)	1.29 (0.30)	−0.03 (−0.07 to 0.02)
Triglycerides (mmol/l)	1.10 (0.80–1.56)	0.01 (−0.03 to 0.05)
*Metabolic and renal markers*		
Fasting plasma glucose (mmol/l)	5.36 (1.27)	−0.17 (−0.21 to −0.13)***
Serum creatinine (µmol/1)	89.7 (21.3)	0.13 (0.09–0.17)***
Estimated GFR (ml/min/1.73 m^2^)	87.0 (17.0)	−0.14 (−0.18 to −0.10)***
*Trace elements*		
Serum zinc (mg/l)	0.94 (0.12)	0.11 (0.07–0.16)***
Serum ionized calcium (mmol/l)	1.18 (0.05)	0.06 (0.02–0.10)**
Serum potassium (mmol/l)	3.92 (0.30)	−0.02 (−0.06 to 0.02)

*BMI* body mass index, *CHD* coronary heart disease, *CI* confidence interval, *DBP* diastolic blood pressure, *GFR* glomerular filtration rate, *HDL*-*C* high-density lipoprotein cholesterol, *IQR* interquartile range, *SD* standard deviation, *SBP* systolic blood pressure

Asterisks indicate the level of statistical significance: * *P* < 0.05; ** *P* < 0.01; *** *P* < 0.001; ^†^Adjusted for age

### Magnesium and risk of fractures

**Fig. 1:**
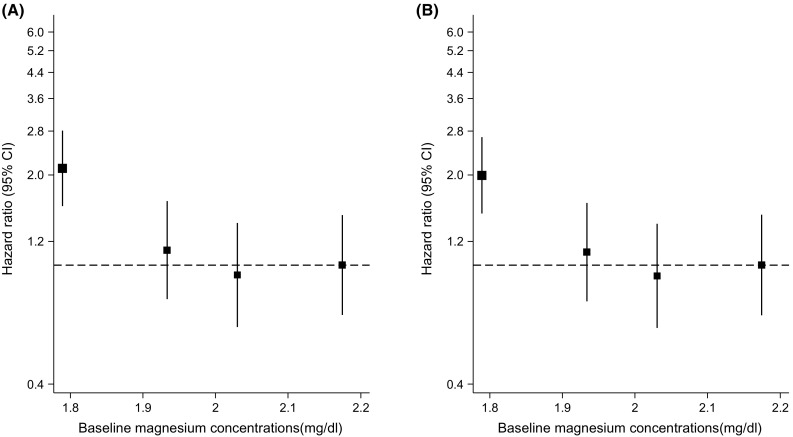
Hazard ratios for incident total fractures by quartiles of serum magnesium levels. **a** Adjusted for age; **b** adjusted for age, body mass index, height, systolic blood pressure, smoking status, history of diabetes, alcohol consumption, and physical activity; the mean magnesium level (mg/dl) was 1.79 for the lowest quartile; 1.93 for the second quartile; 2.03 for the third quartile; and 2.17 for the top quartile; *CI* confidence interval

**Fig. 2:**
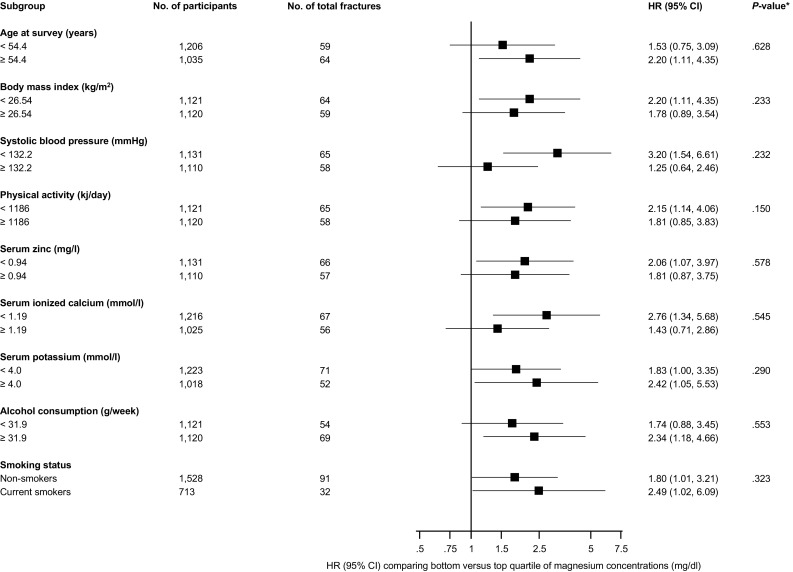
Hazard ratios for serum magnesium levels and total fractures risk by several participant level characteristics. Hazard ratios were adjusted for age, body mass index, height, systolic blood pressure, smoking status, history of diabetes, alcohol consumption, and physical activity; *CI* confidence interval, *GFR* glomerular filtration rate, *HR* hazard ratio; * *P* value for interaction; cut-offs used for age, body mass index, systolic blood pressure, physical activity, serum zinc, serum ionized calcium, serum potassium, and alcohol consumption are median values

During a median (interquartile range) follow-up of 25.6 (17.6–27.9) years, 123 incident total fractures (annual rate 2.49/1000 person-years at risk; 95% CI 2.08–2.97) were recorded. Of the 22 men who had excess serum magnesium concentrations at baseline, none of them experienced a fracture at follow-up; and of the total number of incident fractures, 78 were femoral fractures. In analyses adjusted for several established risk factors (age, body mass index, height, systolic blood pressure, smoking, history of diabetes, alcohol consumption, and physical activity), a non-linear inverse association was observed between serum magnesium and risk of total fractures (Fig. 1). Comparing the bottom quartile versus top quartile of serum magnesium concentrations, the age-adjusted HR for total fractures was 2.10 (95% CI 1.30–3.41; *P* = 0.003), which remained consistent 1.99 (95% CI 1.23–3.24; *P* = 0.005) following further adjustment for several established risk factors. The HR was minimally attenuated to 1.80 (95% CI 1.10–2.94; *P* = 0.019) on additional adjustment for eGFR, SES, total energy intake, serum zinc, serum potassium, and serum ionized calcium (Table 2). The association between serum magnesium and total fractures was not significantly modified by several clinically relevant characteristics (*P* for interaction ≥0.10 for each; Fig. 2). In a sensitivity analysis which was limited to a sample of 2129 men (117 total fractures) after the first 5 years of follow-up was excluded, the associations remained consistent: 2.05 (95% CI 1.25–3.36; *P* = 0.005), 1.95 (95% CI 1.19–3.21; *P* = 0.008), and 1.77 (95% CI 1.07–2.93; *P* = 0.026) respectively.

**Table 2:** Association of serum magnesium and incident fractures by quartiles of serum magnesium

Serum magnesium (mg/dl)	Events/total	Model 1		Model 2		Model 3	
		HR (95% CI)	*P* value	HR (95% CI)	*P* value	HR (95% CI)	*P* value
*Total fractures*							
Q4 (2.08–2.55)	26/557	ref		ref		ref	
Q3 (1.98–2.08)	24/564	0.93 (0.53–1.61)	0.787	0.92 (0.53–1.60)	0.768	0.86 (0.50–1.51)	0.608
Q2 (1.88–1.98)	27/558	1.12 (0.65–1.92)	0.677	1.10 (0.64–1.90)	0.720	1.01 (0.59–1.74)	0.968
Q1 (0.92–1.88)	46/566	2.10 (1.30–3.41)	0.003	1.99 (1.23–3.24)	0.005	1.80 (1.10–2.94)	0.019
*Femoral fractures*							
Q4 (2.08–2.55)	15/556	ref		ref		ref	
Q3 (1.98–2.08)	16/556	1.08 (0.53–2.19)	0.828	1.08 (0.53–2.18)	0.840	1.01 (0.50–2.04)	0.987
Q2 (1.88–1.98)	16/553	1.20 (0.59–2.43)	0.615	1.19 (0.59–2.42)	0.626	1.08 (0.53–2.20)	0.833
Q1 (0.92–1.88)	31/559	2.56 (1.38–4.76)	0.003	2.43 (1.30–4.53)	0.005	2.13 (1.13–3.99)	0.019

*CI* confidence interval, *HR* hazard ratio, *ref* reference, *Q* quartile

Model 1: Adjusted for age, Model 2: Model 1 plus body mass index, height, systolic blood pressure, smoking, history of diabetes, alcohol consumption, and physical activity, Model 3: Model 2 plus estimated glomerular filtration rate, socioeconomic status, total energy intake, serum zinc, serum potassium, and serum ionized calcium

In analyses adjusted for several established risk factors, there was a continuous association of serum magnesium with risk of femoral fractures, which was potentially consistent with either a curvilinear or linear shape (online Appendix 3). The corresponding adjusted HRs for femoral fractures were 2.56 (95% CI 1.38–4.76; *P* = 0.003), 2.43 (95% CI 1.30–4.53; *P* = 0.005), and 2.13 (95% CI 1.13–3.99; *P* = 0.019) respectively.

Based on the distribution of serum magnesium in the current study and published guidelines which suggest a normal reference range of 1.8–2.3 mg/dl [31], HRs for total and femoral fractures were calculated using the following serum magnesium categories: (1) <1.8 mg/dl (group 1 and referent, hypomagnesemia); (2) 1.8–2.0 mg/dl (group 2); and (3) >2.1 mg/dl (group 3). The inverse and independent associations of serum magnesium with the risk of total and femoral fractures remained consistent (Table 3). In subsidiary analyses using the bottom quartile of serum magnesium concentration as a reference comparison, participants in the top quartile of serum magnesium concentrations had a decreased risk of both total and femoral fractures (online Appendix 4).

**Table 3:** Association of serum magnesium and incident fractures by clinical categories of serum magnesium

Serum magnesium(mg/dl)	Events/total	Model 1		Model 2		Model 3	
		HR (95% CI)	*P* value	HR (95% CI)	*P* value	HR (95% CI)	*P* value
*Total fractures*							
>2.0	34/722	ref		ref		ref	
1.8–2.0	74/1387	1.25 (0.83–1.88)	0.284	1.24 (0.82–1.86)	0.308	1.18 (0.78–1.78)	0.431
<1.8	15/136	3.30 (1.79–6.08)	< 0.001	2.95 (1.59–5.49)	0.001	2.51 (1.34–4.70)	0.004
*Femoral fractures*							
>2.0	20/719	ref		ref		ref	
1.8–2.0	48/1370	1.42 (0.84–2.39)	0.192	1.41 (0.83–2.37)	0.202	1.32 (0.78–2.23)	0.304
<1.8	10/135	3.80 (1.77–8.16)	0.001	3.32 (1.53–7.22)	0.002	2.64 (1.20–5.78)	0.016

*CI* confidence interval, *HR* hazard ratio, *ref* reference

Model 1: Adjusted for age, Model 2: Model 1 plus body mass index, height, systolic blood pressure, smoking, history of diabetes, alcohol consumption, and physical activity, Model 3: Model 2 plus estimated glomerular filtration rate, socioeconomic status, total energy intake, serum zinc, serum potassium, and serum ionized calcium

There was no evidence of an association of dietary magnesium intake with risk of total and femoral fractures (Table 4).

**Table 4:** Association of dietary magnesium intake and incident fractures

Dietary magnesium(mg/day)	Events/total	Model 1		Model 2		Model 3	
		HR (95% CI)	*P* value	HR (95% CI)	*P* value	HR (95% CI)	*P* value
*Total fractures*							
Q4 (≥ 457.70)	35/561	ref		ref		ref	
Q3 (413.40–457.60)	29/561	0.80 (0.49–1.31)	0.382	0.85 (0.52–1.39)	0.512	0.92 (0.56–1.52)	0.743
Q2 (372.59–413.32)	29/561	0.77 (0.47–1.26)	0.305	0.82 (0.50–1.34)	0.422	0.88 (0.53–1.46)	0.626
Q1 (30.13–372.53)	30/562	0.82 (0.50–1.34)	0.429	0.82 (0.50–1.34)	0.424	0.89 (0.54–1.47)	0.654
*Femoral fractures*							
Q4 (≥ 457.70)	18/553	ref		ref		ref	
Q3 (413.40–457.60)	17/554	0.93 (0.48–1.80)	0.821	0.98 (0.51–1.91)	0.959	1.08 (0.55–2.12)	0.813
Q2 (372.59–413.32)	23/558	1.17 (0.63–2.18)	0.609	1.25 (0.67–2.32)	0.488	1.34 (0.71–2.52)	0.362
Q1 (30.13–372.53)	20/559	1.07 (0.57–2.02)	0.838	1.04 (0.55–2.00)	0.896	1.20 (0.62–2.32)	0.581

*CI* confidence interval, *HR* hazard ratio, *ref* reference, *Q* quartile

Model 1: Adjusted for age, Model 2: Model 1 plus body mass index, height, systolic blood pressure, smoking, history of diabetes, alcohol consumption, and physical activity, Model 3: Model 2 plus estimated glomerular filtration rate, socioeconomic status, total energy intake, serum zinc, serum potassium, and serum ionized calcium

## Comment

### Summary of findings

We have shown in this population-based prospective study of middle-aged Caucasian men that low serum magnesium concentrations are independently associated with an increased risk of future total and femoral fractures. Whiles serum magnesium was non-linearly associated with risk of total fractures, we observed a continuous decrease in risk of femoral fractures with increasing levels of serum magnesium. Given the low event rate for femoral fractures, further work is required to determine whether a curvilinear or linear shape would better describe the relationship. The associations persisted after controlling for several confounders including other trace elements such as serum zinc and serum ionized calcium. There was also no evidence of effect modification by several clinically relevant subgroups. In a subsidiary analyses, high serum magnesium concentrations were associated with a reduced risk of fractures and we found no evidence of an association of dietary magnesium intake with risk of both total and femoral fractures.

### Comparison with previous work

Previous published articles exploring the prospective association between serum magnesium and risk of fractures are scarce. In the only study published to date to our knowledge, investigators employing the large-scale EPIC cohort were unable to show a statistically significant association between serum magnesium concentrations and risk of fractures after multivariable adjustment [14]. A broad body of evidence suggests that magnesium has a beneficial effect on bone health; however, the greater majority of studies have explored dietary magnesium as an exposure and not serum magnesium status. Although high dietary magnesium intake has been linked with greater bone mineral density [6, 8]; the evidence of a beneficial effect on risk of fractures is not convincing. The majority of studies including a recent pooled analysis of 12 studies have consistently demonstrated that high dietary magnesium intake is not protective of risk of fractures [6, 11]. Indeed, our subsidiary analysis of dietary magnesium and fracture risk showed statistically non-significant associations. In contrast, magnesium consumption in excess of the recommended dietary allowance has also been shown to be associated with an increased risk of lower-arm and wrist fractures in the Women’s Health Initiative Observational Study. The investigators however postulated that this might be due to greater physical activity and risk of increased falls in this group of women [6]. Whether the current findings of an inverse association of serum magnesium with fracture risk reflects a true association may need to be confirmed in other large-scale prospective studies.

### Possible explanations for findings

Though magnesium, the second most abundant intracellular cation, plays a role in several cellular processes; the mechanistic pathways by which magnesium influences bone metabolism are currently unclear. However pathways proposed that link low serum levels of magnesium to increased fracture risk include impaired bone growth, decreased bone density, and bone fragility or osteoporosis [3]. Via a nitric oxide-dependent mechanism, magnesium has an effect on osteoblast activity and number of osteoclasts [5]. In selective dietary magnesium depletion in animal models, Rude and colleagues observed bone loss, decrease in osteoblasts, and increased osteoclasts in these animals. Magnesium deficiency leads to resistance or low vitamin D and parathyroid hormone levels [3, 32–34], which may lead to reduced bone formation and subsequently increased risk of fractures. Magnesium also affects bone metabolism by altering calcium homeostasis [1, 5]. Magnesium deficiency in animal models have been shown to stimulate production of cytokines, which are known to increase osteoclastic bone resorption. An increase in receptor activator of nuclear factor kB ligand (RANKL) and a decrease in osteoprotegerin (OPG), both caused by magnesium deficiency, cause an increase in bone resorption. Magnesium is known to stabilize and slow the transformation of amorphous calcium phosphate to hydroxyapatite [35], which gives bones higher bone mass [36]. Magnesium is essential for normal neurological and muscular function [12]; low serum magnesium concentrations (hypomagnesemia) may manifest as muscle weakness and seizures [37], which lead to falls and subsequently fractures. Several reasons could account for the null findings demonstrated between dietary magnesium and risk of fractures. First, observational studies of dietary intake are prone to misclassification bias in addition to the fact that food sources of magnesium are also frequently high in nutrients such as potassium and calcium; which are beneficial and interact with other micronutrients to maintain bone health and therefore it is difficult to evaluate the separate effect of each nutrient. Second, the possibility that individuals dietary intake of magnesium may not correlate with their total body stores or serum levels. Though a low serum magnesium concentration generally signifies low total body magnesium status; serum magnesium concentrations do not accurately reflect total body magnesium stores [38, 39]. Thus, it is not uncommon to find normal serum magnesium concentrations in the presence of depleted total body stores and vice versa.

### Implications of findings

The long-term prospective and independent association demonstrated between baseline magnesium concentrations and risk of fractures, suggest that serum magnesium status may modify fracture risk and this may have implications for clinical practice. The findings do suggest that avoiding low serum concentrations of magnesium may be a promising though unproven strategy for risk prevention of fractures. Although serum magnesium concentration depends on magnesium intake from food and water, suggesting that increased intake may lead to increased serum levels and reduced fracture risk, this is not supported by the available evidence. Indeed, a number of studies including the present analysis, have shown no correlation between dietary magnesium intake and serum magnesium concentrations [14, 40]. It has been postulated this could be a reflection of the tight homeostatic control of the magnesium cation in the circulation [14]. In addition, it is also known that the absorption and bioavailability of minerals can be affected by interactions between different dietary nutrients; such as the case of fibre, calcium, and phosphorus for magnesium [41, 42]. Serum magnesium concentration is regulated by a balance between intestinal absorption and excretion by the kidneys; therefore, decreased intestinal absorption, increased gastrointestinal loss, or renal loss, may contribute to the lack of a correlation between dietary intake and serum concentrations of magnesium. Factors or conditions known to cause these include old age, inflammatory bowel disorders, malabsorption syndromes, diabetes, certain medications (e.g., diuretics, proton-pump inhibitors), as well as renal impairment [39, 43]. Based on these observations, modulating or treating these underlying co-morbid conditions or factors that predispose to low serum magnesium concentrations may subsequently help to prevent the development of hypomagnesemia. Though hypomagnesemia is common and its prevalence in the general population ranges from 2.5 to 15% [44], most patients are usually asymptomatic and symptoms usually manifest only when the serum concentrations fall below 1.2 mg/dl [45]. Since serum magnesium concentration is not measured as part of the routine blood screening panel, individuals with low serum levels are difficult to identify. Serum magnesium screening should therefore be conducted in the elderly and in situations such as chronic diarrhoea, hypocalcemia, hypokalemia, and other co-morbid conditions that predispose to low circulating levels of magnesium. Magnesium supplementation may also be another way of increasing serum levels, especially for people who are unable to get the recommended dietary allowance from dietary sources. Though the evidence is sparse, a number of human studies as well as experiments in animal models have indeed shown that magnesium supplementation is associated with improvement in bone mineral density and suppression of bone turn over markers [9, 46]. Sojka and Weaver [46] in their 2-year magnesium supplementation study of a group of menopausal women, concluded from their findings that magnesium therapy resulted in greater bone density and appeared to have prevented the risk of fractures. The overall evidence suggests that increasing serum magnesium concentrations may protect against the future risk of fractures; however, well-designed magnesium supplementation trials are needed to investigate these potential therapeutic implications.

### Strengths and limitations

The current analysis employed a large population-based prospective cohort of men who were selected to be nationally representative. The study sample had a high response rate and there was complete follow-up for all participants. To our knowledge, this is the first prospective analysis that shows an independent and inverse association between serum magnesium and incident fractures. Assay measurements for serum magnesium employed atomic absorption spectrometry which has a high level of precision. Other strengths of the current analysis include the long follow-up period of over 20 years and comprehensive analysis with adjustment for a broad panel of lifestyle, nutritional and socioeconomic factors, and biochemical markers enabling reliable assessments of the associations. The research was limited because of the observational nature of the study; the findings cannot be generalized to women and other populations; inability to correct for within-person variability in serum magnesium levels because of absence of data on repeat measurements; absence of data on other types of fractures such as vertebral fractures; absence of data on fractures related to falls or fall-related hospitalizations, given that low serum magnesium status leads to an increased risk of falls; inability to adjust for other potential confounders such as vitamin D, bone mineral density, use of medications, previous fracture, and prevalent conditions such as thyroid disease, crohn’s disease, coeliac disease, and myelomas which are uncommon in the study population; and inability to replicate the results in an independent cohort because this data was not available. In addition, serum magnesium levels does not necessarily reflect total body magnesium content [47]; however, it is the best estimate of magnesium status as it correlates with ionized and intracellular magnesium [48].

## Conclusion

Low serum magnesium concentrations is independently associated with an increased risk of total and femoral fractures in middle-aged Caucasian men. Further research is needed to replicate these results in women and other populations as well as assess the potential relevance of serum magnesium in fracture prevention.

## Electronic supplementary material

Below is the link to the electronic supplementary material.

Supplementary material 1 (DOCX 82 kb)
