# Supplementary material for: Low serum magnesium levels are associated with increased risk of fractures: a long-term prospective cohort study
Source: Eur J Epidemiol. 2017 Apr 12;32(7):593–603. doi: 10.1007/s10654-017-0242-2 (PMC5570773; doi:10.1007/s10654-017-0242-2)
Supplement: Supplementary file 1 — Supplementary material 1 (DOCX 82 kb) [file 10654_2017_242_MOESM1_ESM.docx]

**APPENDIX SUPPLEMENTS**

**Low serum magnesium levels are associated with increased risk of fractures: A long-term prospective cohort study**

| **Appendix 1** | STROBE 2007 Statement—Checklist of items that should be included in reports of cohort studies |
| --- | --- |
| **Appendix 2** | Baseline participant characteristics by quartiles of serum magnesium |
| **Appendix 3** | Hazard ratios for incident femoral fractures by quartiles of serum magnesium levels |
| **Appendix 4** | Association of serum magnesium and incident fractures by quartiles of serum magnesium (with quartile 1 as a reference comparison) |

**Appendix 1:** STROBE 2007 Statement

| **Section/Topic** | Item # | Recommendation | Reported on page # |
| --- | --- | --- | --- |
| **Title and abstract** | 1 | (*a*) Indicate the study’s design with a commonly used term in the title or the abstract | Page 1 |
|  |  | (*b*) Provide in the abstract an informative and balanced summary of what was done and what was found | Page 2 |
| Introduction | | |  |
| Background/rationale | 2 | Explain the scientific background and rationale for the investigation being reported | Page 3-4 |
| Objectives | 3 | State specific objectives, including any prespecified hypotheses | Page 3-4 |
| Methods | | |  |
| Study design | 4 | Present key elements of study design early in the paper | Study Design and Participants |
| Setting | 5 | Describe the setting, locations, and relevant dates, including periods of recruitment, exposure, follow-up, and data collection | Study Design and Participants |
| Participants | 6 | (*a*) Give the eligibility criteria, and the sources and methods of selection of participants. Describe methods of follow-up | Study Design and Participants |
|  |  | (*b*) For matched studies, give matching criteria and number of exposed and unexposed | Not applicable |
| Variables | 7 | Clearly define all outcomes, exposures, predictors, potential confounders, and effect modifiers. Give diagnostic criteria, if applicable | Risk Factor Assessment |
| Data sources/ measurement | 8* | For each variable of interest, give sources of data and details of methods of assessment (measurement). Describe comparability of assessment methods if there is more than one group | Risk Factor Assessment |
| Bias | 9 | Describe any efforts to address potential sources of bias | Statistical Analyses |
| Study size | 10 | Explain how the study size was arrived at | Statistical Analyses |
| Quantitative variables | 11 | Explain how quantitative variables were handled in the analyses. If applicable, describe which groupings were chosen and why | Statistical Analyses |
| Statistical methods | 12 | (*a*) Describe all statistical methods, including those used to control for confounding | Statistical Analyses |
|  |  | (*b*) Describe any methods used to examine subgroups and interactions | Statistical Analyses |
|  |  | (*c*) Explain how missing data were addressed | Not applicable |
|  |  | (*d*) If applicable, explain how loss to follow-up was addressed | Not applicable |
|  |  | (*e*) Describe any sensitivity analyses | Statistical Analyses |
| Results | | |  |
| Participants | 13* | (a) Report numbers of individuals at each stage of study—eg numbers potentially eligible, examined for eligibility, confirmed eligible, included in the study, completing follow-up, and analysed | Study population |
|  |  | (b) Give reasons for non-participation at each stage | Study population |
|  |  | (c) Consider use of a flow diagram |  |
| Descriptive data | 14* | (a) Give characteristics of study participants (eg demographic, clinical, social) and information on exposures and potential confounders | Results; Table 1; Appendix 2 |
|  |  | (b) Indicate number of participants with missing data for each variable of interest |  |
|  |  | (c) Summarise follow-up time (eg, average and total amount) | Results |
| Outcome data | 15* | Report numbers of outcome events or summary measures over time | Results |
| Main results | 16 | (*a*) Give unadjusted estimates and, if applicable, confounder-adjusted estimates and their precision (eg, 95% confidence interval). Make clear which confounders were adjusted for and why they were included | Results; Tables 2-4 |
|  |  | (*b*) Report category boundaries when continuous variables were categorized | Results; Tables 2-4 |
|  |  | (*c*) If relevant, consider translating estimates of relative risk into absolute risk for a meaningful time period |  |
| Other analyses | 17 | Report other analyses done—eg analyses of subgroups and interactions, and sensitivity analyses | Results; Figure 2 |
| Discussion |  |  |  |
| Key results | 18 | Summarise key results with reference to study objectives | Discussion - Summary of main findings |
| **Limitations** |  |  |  |
| Interpretation | 20 | Give a cautious overall interpretation of results considering objectives, limitations, multiplicity of analyses, results from similar studies, and other relevant evidence | Discussion |
| Generalisability | 21 | Discuss the generalisability (external validity) of the study results | Discussion |
| Other information |  |  |  |
| Funding | 22 | Give the source of funding and the role of the funders for the present study and, if applicable, for the original study on which the present article is based | Page 16 |

**Appendix 2.** Baseline participant characteristics by quartiles of serum magnesium

|  | **Quartile 1**  **Mean (SD), median (IQR), or n (%)** | **Quartile 2**  **Mean (SD), median (IQR), or n (%))** | **Quartile 3**  **Mean (SD), median (IQR), or n (%)** | **Quartile 4**  **Mean (SD), median (IQR), or n (%)** | ***P*-value** |
| --- | --- | --- | --- | --- | --- |
| Magnesium (mg/dl) | 1.79 (0.09) | 1.93 (0.03) | 2.03 (0.3) | 2.17 (0.08) | < 0.0001 |
|  |  |  |  |  |  |
| ***Questionnaire/Prevalent conditions*** |  |  |  |  |  |
| Age at survey (years) | 53.1 (5.5) | 52.4 (5.7) | 53.3 (4.8) | 53.7 (3.9) | 0.0002 |
| Alcohol consumption (g/week) | 86.8 (170.8) | 78.9 (154.8) | 71.6 (101.1) | 66.0 (110.3) | 0.063 |
| Total energy intake, kJ/day | 9,998 (2,736) | 9,900 (2,676) | 9,871 (2,557) | 9,647 (2,450) | 0.143 |
| Socioeconomic status | 8.70 (4.09) | 8.46 (4.24) | 8.60 (4.30) | 8.26 (4.33) | 0.339 |
| Dietary magnesium intake (mg/day) | 419.4 (72.2) | 419.6 (69.7) | 417.2 (64.3) | 412.3 (70.8) | 0.266 |
| History of diabetes |  |  |  |  |  |
| No | 527 (93.1) | 534 (95.7) | 551 (97.7) | 544 (97.7) |  |
| Yes | 39 (6.9) | 24 (4.3) | 13 (2.3) | 13 (2.3) | < 0.001 |
| Smoking status |  |  |  |  |  |
| Other | 384 (67.8) | 389 (69.7) | 374 (66.3) | 382 (68.6) |  |
| Current | 182 (32.2) | 169 (30.3) | 190 (33.7) | 175 (31.4) | 0.665 |
| History of hypertension |  |  |  |  |  |
| No | 387 (68.4) | 387 (69.4) | 394 (69.9) | 396 (71.1) |  |
| Yes | 179 (31.6) | 171 (30.7) | 170 (30.1) | 161 (28.9) | 0.796 |
|  |  |  |  |  |  |
| ***Physical measurements*** |  |  |  |  |  |
| BMI (kg/m^2^) | 27.2 (3.8) | 26.7 (3.5) | 27.0 (3.5) | 26.9 (3.5) | 0.209 |
| Height (cm) | 173.0 (6.1) | 172.9 (6.3) | 172.9 (6.3) | 172.5 (6.2) | 0.560 |
| SBP (mmHg) | 134 (17) | 134 (16) | 134 (17) | 134 (18) | 0.931 |
| DBP (mmHg) | 89 (11) | 88 (10) | 89 (10) | 88 (11) | 0.513 |
| Physical activity (kj/day) | 1,500 (1,414) | 1,596 (1,658) | 1,547 (1,370) | 1,540 (1,502) | 0.760 |
|  |  |  |  |  |  |
| ***Lipid markers*** |  |  |  |  |  |
| Total cholesterol (mmol/l) | 5.82 (1.13) | 5.82 (1.10) | 5.93 (1.02) | 6.06 (1.05) | 0.0002 |
| HDL-C (mmol/l) | 1.31 (0.33) | 1.29 (0.29) | 1.29 (0.28) | 1.28 (0.29) | 0.485 |
| Triglycerides (mmol/l) | 1.08 (0.79-1.53) | 1.08 (0.79-1.52) | 1.11 (0.80-1.57) | 1.12 (0.83-1.64) | 0.118 |
|  |  |  |  |  |  |
| ***Metabolic and renal markers*** |  |  |  |  |  |
| Fasting plasma glucose (mmol/l) | 5.73 (1.96) | 5.25 (0.96) | 5.24 (0.79) | 5.22 (0.94) | < 0.0001 |
| Serum creatinine (µmol/1) | 87.2 (15.3) | 89.1 (14.3) | 89.9 (12.5) | 92.7 (34.8) | 0.0002 |
| Estimated GFR (ml/min/1.73 m^2^) | 90.1 (17.7) | 87.7 (15.7) | 86.1 (19.0) | 84.0 (15.7) | < 0.0001 |
|  |  |  |  |  |  |
| ***Trace elements*** |  |  |  |  |  |
| Serum zinc (mg/l) | 0.92 (0.13) | 0.94 (0.12) | 0.94 (0.11) | 0.95 (0.12) | 0.0006 |
| Serum ionized calcium (mmol/l) | 1.18 (0.05) | 1.18 (0.05) | 1.18 (0.05) | 1.18 (0.05) | 0.016 |
| Serum potassium (mmol/l) | 3.93 (0.28) | 3.93 (0.37) | 3.92 (0.27) | 3.90 (0.29) | 0.171 |

BMI, body mass index; CHD, coronary heart disease; CI, confidence interval; DBP, diastolic blood pressure; GFR, glomerular filtration rate;

HDL-C, high-density lipoprotein cholesterol; IQR, interquartile range; SD, standard deviation; SBP, systolic blood pressure;

**Appendix 3.** Hazard ratios for incident femoral fractures by quartiles of serum magnesium levels

**A**, adjusted for age; **B**, adjusted for age, body mass index, height, systolic blood pressure, smoking status, history of diabetes, alcohol consumption, and physical activity; the mean magnesium level (mg/dl) was 1.79 for the lowest quartile; 1.93 for the second quartile; 2.03 for the third quartile; and 2.17 for the top quartile; CI, confidence interval

**Appendix 4.** Association of serum magnesium and incident fractures by quartiles of serum magnesium (with quartile 1 as reference comparison)

| **Serum magnesium(mg/dl)** | **Events/**  **Total** | **Model 1** |  | **Model 2** |  | **Model 3** |  |
| --- | --- | --- | --- | --- | --- | --- | --- |
|  |  | HR (95% CI) | *P-*value | HR (95% CI) | *P-*value | HR (95% CI) | *P-*value |
| **Total fractures** | | | | | | | |
| Q1 (0.92-1.88) | 46 / 566 | ref |  | ref |  | ref |  |
| Q2 (1.88-1.98) | 27 / 558 | 0.53 (0.33 to 0.86) | 0.010 | 0.55 (0.34 to 0.89) | 0.015 | 0.56 (0.35 to 0.91) | 0.019 |
| Q3 (1.98-2.08) | 24 / 564 | 0.44 (0.27 to 0.72) | 0.001 | 0.46 (0.28 to 0.76) | 0.002 | 0.48 (0.29 to 0.79) | 0.004 |
| Q4 (2.08-2.55) | 26 / 557 | 0.48 (0.29 to 0.77) | 0.003 | 0.50 (0.31 to 0.82) | 0.005 | 0.56 (0.34 to 0.91) | 0.019 |
| **Femoral fractures** | | | | | | | |
| Q1 (0.92-1.88) | 31 / 559 | ref |  | ref |  | ref |  |
| Q2 (1.88-1.98) | 16 / 553 | 0.47 (0.26 to 0.86) | 0.014 | 0.49 (0.27 to 0.90) | 0.022 | 0.51 (0.27 to 0.94) | 0.031 |
| Q3 (1.98-2.08) | 16 / 556 | 0.42 (0.23 to 0.77) | 0.005 | 0.44 (0.24 to 0.81) | 0.009 | 0.47 (0.26 to 0.87) | 0.017 |
| Q4 (2.08-2.55) | 15 / 556 | 0.39 (0.21 to 0.73) | 0.003 | 0.41 (0.22 to 0.77) | 0.005 | 0.47 (0.25 to 0.88) | 0.019 |

CI, confidence interval; HR, hazard ratio; ref, reference; Q, quartile

Model 1: Adjusted for age

Model 2: Model 1 plus body mass index, height, systolic blood pressure, smoking, history of diabetes, alcohol consumption, and physical activity

Model 3: Model 2 plus estimated glomerular filtration rate, socioeconomic status, total energy intake, serum zinc, serum potassium, and serum ionized calcium
